# Literary Recreation

**Published:** 1881-04

**Authors:** E. G. Betty


### LITERARY RECREATION.

E. G. Betty, D. D. S.

The sweet and gentle Isaac D'Israeli tells us that "among the Jesuits it was a standing rule of the order, that after an application to study for two hours, the mind of the student should be unbent by some relaxation, however trifling."

He takes this for a text, and preaches us a delightful little sermon, illustrating with choice bits from " the lives of great men," which reminds us we can make our lives, if not exactly sublime, at least less prosy and more enjoyable.

Tycho Brahe polished spectacle glasses; Descartes cultivated a little garden; Richelieu was given to violent exercises, and " was once discovered jumping with his servant, to try who could reach the highest side of a wall." These examples could be multiplied *ad infinitum*, but will suffice. Independently of their interest as literary curiosities, they teach a lesson worthy of emulation. There is no product of a people's history and civilization of so much value as its literature. 'Twas produced by many minds, with many ideas; in its manifold phases variety and wondrous, there is invariably material enough to furnish every shade of intellect with abundant thought-food. Perhaps the most interesting thing in and concerning literature is its own history, and how well it has been written. Bayle, Dr. Johnson, Isaac D'Israeli, Taine, Hazlitt, Duyckinck, and, quite lately, Professor Tyler, of Ann Arbor, together with others, have gathered into a comparatively small compass all the stores of literary wealth. In them we have the biographies boiled down to reasonable limits; while the criticism is from time to time embellished with quotations and extracts from the authors themselves. In the aggregate they form a fund from which we can draw at sight, always assured that the draft will be honored. When read, digested and assimilated, they furnish the mind an inexhaustible source of pleasant and profitable thought. In these histories, be they philosophical, " pastoral-comical, historical-pastoral, tragical-historical, tragical-comical-historical-pastoral," the student and practitioner cannot fail but find the recreation that is to refresh and delight his soul. From the wearying and monotonous labor of the day we can turn to literature for relaxation, amusement. It is never found want ing, be we never so critical or fastidious. From its pure and limpid waters, as from those of the crystal spring, the thirsty soul partakes of the refreshing draughts that make life sweet and give us a foretaste of that happiness the mind pictures for the illimitable future. It is not the present purpose to enter into a learned and philosophical disquisition upon the history of literature in any country; nor a metaphysical inquiry into the character and manifestations of genius; nor a psychological analysis of any author, through the medium of his productions. That we leave for the reader to study in Buckle, Draper, Guizot, and shall be content merely to name a few of the classical works in and for which our language is so rich and justly famous. In several of the authors first mentioned, the matter is, to a greater or lesser extent, fragmentary, and may be taken up and read with profit in odd moments. Notably, and, to the writer's mind, preeminent among them stands the "blessed Jew," Isaac D'Israeli. His "Curiosities of Literature" is a library in itself, scarcely an item, marvellous, rare, curious or quaint, pertaining to the republic of letters, but has been clothed by him in language at once easy-flowing, polished and pure. As Dr. Johnson said of Goldsmith, so we say of D'Israeli, "a man who had the art of being minute without tediousness, and generally without confusion; exact without restraint; and easy without weakness." Inspection will find him all that the curious and inquisitive can desire. His pages not only contain a wonderful variety of subjects, but he brings to their treatment that richness of thought and fertility of resource only seen in large minds, well stored with knowledge. Unlike the recluse, who reads solely for his own enjoyment, D'Israeli has collected a vast store of information, and embellished it with the beauty of his own mind, in a manner that fascinates and entertains, while it conveys a wealth of knowledge truly wonderful. From Taine we have received a history unlike any hitherto produced. It proceeds in a regular order of development from the sources of the English tongue, down through the course of history, coinciding, step by step, with the evolution of thought resulting in the present civilization. Taine's "History of English Literature" and Greene's "Short History of the English People" can with great propriety be studied together, and from this combination one can attain a very fair estimate of the English mind. The critical literature of our language is of too vast extent to permit anything like comprehensive reading, for the man who follows a profession. His time is necessarily engrosed with his practice and its literature. These occupy the greater share of his attention, leaving but little room or opportunity for other things. For this reason will he find, in the authors named, that material which he can appropriate during leisure moments. A few minutes now and then with some charming writer will do a great deal to relieve the tedium of every-day work. The mind will not only acquire good information, but a culture and toning, refining life. Who is there that does not love poor Goldsmith? And for what? It is for his warm-hearted Irish generosity, his weaknesses, kindness, the innate refinement of his heart and mind, above all, the many beauties he has bequeathed us. Washington Irving's "Goldsmith" is but a just tribute to the memory of "poor Goldy," and was written to counteract the baleful influence of that "prince of biographers," and we may add, hero-worshippers, Boswell. Goldsmith's poetry is a model of the capabilities and power of the English language. His numbers glide along like the waters of a brook, fllling our ears not only with "touches of sweet harmony," but a continuous strain of dulcet music. His prose is not only symmetrical, but it, too, is musical. It is in almost every sense a perfect example of the depth, quality and resource of the language. It is not a little to the credit of the great Dr. Johnson that he continually defended his friend Goldsmith against the slurs and calumnies of Boswell. His own genius enabled him to discern in the hapless prodigal those high qualities and pure aspirations the myopic mind of the biographer could neither grasp nor comprehend. 'Tis to the study of Goldsmith and Kirke White that our good friend Dr. Watt no doubt owes his command of language and facility of expression. Language and the power to use it distinguishes man from the brute. We have many masters worthy of study. Over and above the enjoyment we experience in perusing their pages, we absorb knowledge, and gradually acquire the ability to express our own ideas in appropriate and graceful terms.

Of our Irving we may justly be proud. He has not only illuminated American literature by his genius, but he has added lustre to that of the language. A pure, gentle soul he was—one whose heart could truly love and appreciate the noble-minded author of the "Traveller" and the "Deserted Village." What has been said of Goldsmith may, with almost equal propriety, be repeated of Irving. He has been called the "Goldsmith of America," a term, by the way, to which we object, on general principles, not only in this instance, but in every one where an American author is compared with an Englishman. Of course our habits, language, law, etc., are largely derived from the English precedent; but it is eminently unjust to suppose that there is nothing original in the American mind. On the contrary, we are the most original and self-sustaining people amongst civilized nations. On this ground we object to these literary comparisons—Irving the "Goldsmith" of America, Cooper the "Scott," Emerson the "Carlyle," etc. The "Sketch Book" and "Knickerbocker" are familiar as household words to all. What need of eulogium or comment? When "Alhambra" is mentioned, what a train of pleasant thoughts the very name suggests! The legends of the Moor and Spaniard read like a fairy tale of the East; indeed it was from there the Moor brought them. They bring to mind the stories of enchantment in the "Arabian Nights" that so wonderfully impressed the mind during boyhood. The "Tales of a Traveller," "Bracebridge Hall," "Wolfert's Roost," all, with many more, are told in a most felicitous and happy manner. The space alloted this sketch precludes the possibility of enlarging upon a topic so interesting, profitable and seductive. D'Israeli, Goldsmith and Irving have, for some unaccountable reason, always been associated together in the writer's mind. To mention one is equivalent to naming the three. They are a triad than whom 'tis difficult to find a better, purer and more noble. For that reason, as well as the intrinsic merit of their productions, they have become the theme of this paper. To conclude with a quotation; for Emerson says it is a good thing to quote; let his authority be the excuse. "There are writers who have so ministered to our enjoyment as to become associated with our happiest literary recollections. The companionship of their works has been to us as that of an entertaining and cherished friend, whose converse cheers the hour of langour, and brightens the period of recreative pleasure."

